# Quantifying hierarchy and prestige in US ballet academies as social predictors of career success

**DOI:** 10.1038/s41598-023-44563-z

**Published:** 2023-10-30

**Authors:** Yessica Herrera-Guzmán, Alexander J. Gates, Cristian Candia, Albert-László Barabási

**Affiliations:** 1 Centro de Investigación en Complejidad Social (CICS), Facultad de Gobierno, Universidad del Desarrollo, Santiago, 7610658 Chile; 2 School of Data Science, University of Virginia, Charlottesville, VA 22904 USA; 3 Computational Research in Social Science Laboratory, Instituto de Data Science, Facultad de Ingeniería, Universidad del Desarrollo, Santiago, 7610658 Chile; 4 Northwestern Institute on Complex Systems, Northwestern University, Evanston, IL 60208 USA; 5 Network Science Institute, Northeastern University, Boston, MA 02115 USA; 6 Department of Medicine, Brigham and Women’s Hospital, Harvard Medical School, Boston, MA 02115 USA; 7 Department of Network and Data Science, Central European University, Budapest, 1051 Hungary

**Keywords:** Computational science, Scientific data

## Abstract

In the recent decade, we have seen major progress in quantifying the behaviors and the impact of scientists, resulting in a quantitative toolset capable of monitoring and predicting the career patterns of the profession. It is unclear, however, if this toolset applies to other creative domains beyond the sciences. In particular, while performance in the arts has long been difficult to quantify objectively, research suggests that professional networks and prestige of affiliations play a similar role to those observed in science, hence they can reveal patterns underlying successful careers. To test this hypothesis, here we focus on ballet, as it allows us to investigate in a quantitative fashion the interplay of individual performance, institutional prestige, and network effects. We analyze data on competition outcomes from 6363 ballet students affiliated with 1603 schools in the United States, who participated in the Youth America Grand Prix (YAGP) between 2000 and 2021. Through multiple logit models and matching experiments, we provide evidence that schools’ strategic network position bridging between communities captures social prestige and predicts the placement of students into jobs in ballet companies. This work reveals the importance of institutional prestige on career success in ballet and showcases the potential of network science approaches to provide quantitative viewpoints for the professional development of careers beyond science.

## Introduction

Quantifying the processes and behaviors through which some individuals attain success in creative careers is challenging due to multiple factors, including the subjective valuation of creative performance, the multifaceted ways in which success can become manifest through recognition^1^, and data scarcity^2^. However, the recent proliferation of large digital databases capturing many aspects of scientific careers has fueled advances in data-driven methodological tools to capture career and collaboration patterns, productivity, and impact in science. For instance, the field of *science of science*^3^ has unveiled the random impact rule governing the timing of a researchers’ most consequential publication^4^, how authorship team composition influences productivity patterns and impact^5–7^, the enduring influence of scientific advancements, technological innovations, and cultural products^8–10^, and the tracking of scientific careers and hierarchy on the faculty job market^11,12^, to name a few. The extension of these methods from science to other creative domains has advanced the understanding of the dynamics of artist careers. For instance, recent works elucidate individual transition patterns towards high-impact work^13^, the collective impact of substantial works on long-term success^14^, and the role of luck and individual ability as career success driver^15^.

In both scientific and artistic careers, where performance is subjectively appreciated, career success is strongly influenced by social prestige and visibility^1,16,17^. This suggests that artists’ career success is highly dependent on their social networks and prestige. Previous research implementing quantitative tools from science of science and network science demonstrate the usefulness of these tools to map how social networks shape cultural endeavors^18^. For example, structural properties of teams and collaboration networks in performing arts are strong predictors of artists’ productivity^19^. In addition, network analysis suggests that the position of artists or teams in social networks plays an essential role in allocating resources and rewards and predicts future impact^20,21^, brokerage in collaboration networks can boost individual creativity of performing artists^22^, and the professional network position and rich-get-richer mechanism drives the allocation of acting jobs^23^. These findings support the fact that network position and social prestige are strong predictors of career success in artistic professions. Yet, due in large part to the subjectivity in the valuation of performance quality, many difficulties remain in quantitatively disentangling the contributions of individual performance and collective patterns of social influence on success in creative professions.

To address the relationship between individual performance, institutional prestige, and career outcomes, here we focus on *ballet*, an influential performing art with a long history dating back to the 17th century, when it was promoted by King Louis XIV as a display of the elegance, power, and perfection of human beings^24^. During this period, the access of dancers to the royal court required artistic talent and physical abilities, yet it was significantly facilitated by their membership in a guild or by access to the king, who could grant the privilege by royal decree. Likewise, in the modern era, ballet performance is strongly dependent on physical abilities^25^, which are shaped by perfectionist tendencies^26–28^ and inhibited by physical stressors like injuries, over-training, eating disorders, and poor sleep behaviors^29–32^. The combination of these factors can undoubtedly impact the further development and longevity of dancers’ professional career^33^. At the same time, success in ballet is also recognized as highly dependent on physical attributes^34^, specific personality traits^35–37^, and may also be influenced by subjective perceptions of quality and previous experiences in performing arts^38,39^. This centralizes the social system in ballet, consisting of the network of relationships and hierarchies between dancers, schools, companies, and all other members of the ballet community, which preserves the historical tradition of controlling access to prestigious institutions and professional connections that could ultimately play an essential role in promoting dancers’ success. However, there is a lack of systematic research to quantify the effects of social network connections and prestige on the career success in ballet. Hence, by investigating the social drivers of success in ballet, we do not only inform about the social mechanisms of this performing art and our cultural heritage, but also can directly test the tools of *science of science* in another creative domain.

Our research delves deep into the complex world of the ballet academic system and its relationship with social prestige and career success. While awards and high achievement are undoubtedly crucial in attaining social recognition^40–42^, we propose the use of network centrality as a more precise indicator of social prestige, as it underscores the critical role of social connections in enhancing prestige^43^. We hypothesize that the prestige of a school facilitates the professional development and job placement of its students, which ultimately elevates the school’s external prestige measured by the number of professional dancers they produce, something that has been observed in other creative fields^12,21,44,45^. Thus, dancers may leverage this principle by affiliating with prestigious ballet academies that provide access to a larger network of dance professionals promoting talented dancers.

As a proxy for dance performance, we use competition outcomes of over 6000 young dancers competing at the Youth America Grand Prix (YAGP; for more details see SI, Section S1) from 2000 to 2021. The YAGP competition system filters the participants to the most promising dancers, hence providing a unique opportunity to capture the desired technical and artistic attributes in the ballet market based on jury assessment. The YAGP awards competition medals (gold, silver, and bronze) based on technical and artistic proficiency; and the *Grand Prix*, an award based on the subjective appreciation of the jury. Although multiple biases in performing arts competitions are possible^46^, medals and awards have been long used as an objective metric of performance in different domains^47–50^. Thus, the YAGP competition outcomes represent an objective instrument derived from an efficient system of expert’s opinion evaluating ballet performance.

Using the YAGP data, we build the network of ballet academies from their students participation in the competition and create a ranking of ballet academies by their betweenness centrality, which functions as a validated network-based indicator of prestige. Next, we align students’ competition outcomes with the academic ranking of their affiliations to predict the job placement of ballet students. Overall, our analysis unveils the ballet pre-professional landscape by underscoring the critical role of school prestige in the selection of dancers, even at an equal proficiency level of performance.

Ultimately, our research broadens the scope of the *science of science* methodologies to the performing arts, empowering us to identify the impact of institutions on the young dancer’s careers. This research also contributes to understanding the multifaceted influences of social prestige on career success. Within ballet, the quantitative understanding of network influences on dancer success may also inform equitable policies for auditions and affirmative action that can support a fairer evaluation of candidates in the ballet industry and other professional areas where creative performance is essential. To the best of our knowledge, our study is the first attempt to systematically investigate the effect of social drivers on success in ballet, and contributes to the general understanding of the social contexts driving human creativity, that also broaden the understanding of the evolution of performing arts and our cultural heritage.

## Results

### Network of ballet schools

The Youth America Grand Prix (YAGP) competition plays a pivotal role in supporting young ballet students by fostering connections with a network of dance professionals and academies of international presence. Our hypothesis is that the systematic positioning of schools as top contenders in the competition establishes a hierarchical prestige within the network of ballet schools. This prestige, derived from competition outcomes, subsequently impacts the social system of the ballet industry, leading to a more systematic distribution of awards, job placements, and resources such as scholarships for attracting talented students.

Theories of social stratification vary in their arguments about the formation of social prestige, yet one dominant theme is achievement, conceptualized as a source of social stratification and hierarchical order^41^. Metrics related to achievement (e.g. citations, awards, fellowships, honorary degrees, grants) have been used to understand the role of prestige for career success in academia^3^ and faculty hiring^12^. On the other hand, the implied hierarchical differences among individuals in a social context can be captured by network metrics, like network position or connectedness, which are useful as indirect measures of social prestige^43^. For instance, research on the visual arts has demonstrated that network position objectively captures social prestige and is a good predictor of career success^21^. Grounded in this approach, we construct the network of ballet academies from the YAGP data and create a network-based ranking using each school’s centrality, a key contribution of this paper.

In the YAGP co-competition network, schools are represented as nodes, and a link is established between two schools if their students were ranked among the top 12 in the same competition venue. This network comprises 1603 ballet schools and 55,778 links, providing a comprehensive representation of the ballet academy ecosystem (details on the organization of ballet schools in the U.S. can be found in SI, Section S2) The co-competition network is constructed from both the multiple regional semi-finals and yearly finals competition stages. Thus, the link between two schools captures that both schools were able to produce top dancers under the same competition setting and reflects a degree of similarity in training quality. Connectivity within the co-competition network thus forms an ordered hierarchy in which schools’ high achievement contributes to social prestige which is then directly perceived by others^40–42^. Specifically, highly connected schools in the co-competition are more likely to repeatedly have top dancers in the competition relative to their less connected counterparts. At the same time, these dancers competed against many different schools, thereby increasing their schools’ visibility within the community, as opposed to schools who only competed against the same subset of competitors.

**Figure 1:**
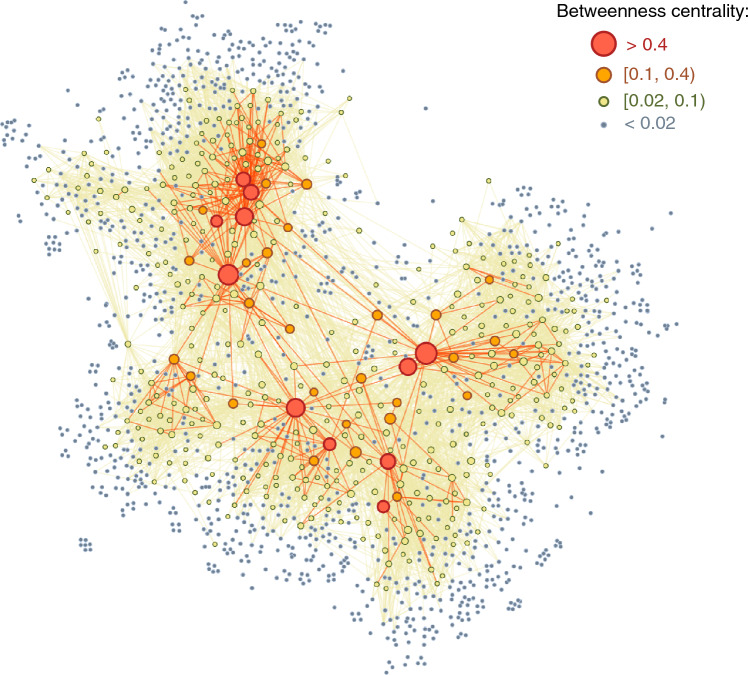
Network of ballet schools. Each node is a ballet school, and two schools are connected if they obtained a top student in the same competition venue. Node size and color reflect schools’ normalized betweenness centrality, $B_k$. Node position is determined by the force-directed graph layout with force estimation $\theta =0.5$ that emphasizes the separation of nodes into clusters. The weak structure (in yellow, network backbone with $\alpha =0.4$) shows dense connectivity within network clusters and sparser connections between clusters and to the periphery; the strong structure (in red, network backbone with $\alpha =0.01$), comprises 166 nodes (10%) and 384 edges (0.6%). This network representation illustrates schools’ hierarchical structure explaining the role of network position for social prestige.

Finally, we capture network effects in the perception of prestige by noting that visibility of ballet schools is further influenced by their potential to bridge between communities in the network. We quantify the bridging capacity, and thus the schools’ social prestige, by the betweenness centrality in the co-competition network. Betweenness centrality is computed for each node, *k*, based on the sum of all-pairs shortest paths which pass through that node:

1 $$\begin{aligned} B_k = \sum _{\sigma (a, b) \in K} \frac{\sigma (a, b|k)}{\sigma (a, b)} \end{aligned}$$

where $\sigma (a, b)$ denotes the number of shortest (*a*, *b*)-paths, and $\sigma (a, b|k)$ is all the shortest paths passing through node *k*^51^. To visually capture the role of betweenness centrality in the network structure, we extract the multi-scale network backbone^52^. This method uses a parameter $\alpha$ for the probability of the existence of an edge and reduces the network to the most fundamental structures and hierarchies based on multi-scale interactions and their relative relevance for the network topology. The resulting network is shown in Fig. 1, where we observe that most schools only attain regional success captured by their low betweenness and weak connections ($\alpha =0.4$, in yellow edges). On the other hand, the network’s strong edges ($\alpha =0.01$, red edges) connects 166 nodes (10% from the total network), forming a core backbone of ballet schools in strategic positions to gain national attention and prestige.

To validate the use of betweenness centrality within the YAGP co-competition network as a proxy for social prestige of ballet schools, we compared the network ranking to a selection of top schools as identified by leading ballet experts. Ballet experts offer comprehensive understanding of dance and the ballet ecosystem, and are widely recognized for their long-standing existence and influence within the dance community in the United States (see SI, Section S2.3 for further details). Here, we aggregate a list of Top Ballet Schools selected by *Dance Magazine* and the highly regarded blog *A Ballet Education*, in total capturing the top 60 ballet schools in the United States (List shown in SI, Fig. S3). We then quantify the extent to which the most prestigious schools as ranked by betweenness centrality recovers the experts’ opinions using the AUC, or the probability that our measure ranks a school listed in the Top Ballet Schools higher than a school not on that list. The AUC is a score between 0 and 1, where a value closer to 1 indicates a probability of correct classification, while a score close to 0.5 indicates that the model performs no better than random guessing. We find an AUC of 0.75 indicating a fair alignment between betweenness centrality and experts’ assessment of the social prestige of ballet schools. Further, betweenness centrality performs better than simpler measures of school prestige and achievement, including the ratio of winning awards or co-competition degree (see SI, Section S2.3). While achievement is certainly a crucial factor in attaining social recognition, our findings suggest that betweenness centrality offers a more accurate measure of social prestige, as it captures the critical interplay between social connections and prestige.

As a whole, our results provide evidence that key network patterns, such as bridging between communities, is closely related with school’s social prestige in the YAGP co-competition network. These findings highlight the utility of network analysis in understanding the relationship between achievement and social prestige.

### Career success of ballet dancers

The hiring process for ballet dancers is limited in opportunities and influenced by a variety of factors including training technique, technical mastery, artistic ability, and even demographics. However, we argue that social prestige plays a significant role beyond performance or ability in predicting the success of dancers’ careers.

To understand the influence of social prestige on a successful job placement, we align the aggregated competition outcomes from 6393 students within the professional age range to the highlighted jobs reported in the *Success Stories* by the YAGP. In total, 385 (6%) young YAGP alumni received a job placement in a dance company. Surprisingly, 22% of YAGP alumni with a job placement did not receive any award in the competition while 10% won in both the semi-finals and finals, and 9% was a finalist but was awarded only in the semi-finals. Moreover, the majority of dancers who received a job placement (59%) won at least one award in the semi-finals but did not advance to the finals. This breakdown suggests that there are different routes and factors other than achievement driving the selection of dancers towards a job placement in a ballet company.

To investigate the intertwined effect of individual achievement and social prestige on job placements, we build a logistic regression model to predict which students are placed into a dance company job. Our dependent variable is success *S*, measured as a binary outcome, where $S_i = 1$ if student *i* obtained a job placement in a ballet company and $S_i = 0$ otherwise. The independent variables include the aggregated measures of students’ achievement within the YAGP competition, such as total awards by type ($Gold_i$, $Silver_i$, $Bronze_i$, and $GrandPrix_i$) and total number of competitions ($Competitions_i$), as well as the normalized and re-scaled schools’ betweenness centrality measure for social prestige ($Prestige_k$). To control for potential confounding factors, we also include a control variable of the student’s gender ($Gender_i$). Our primary model is specified as follows:

2 $$\begin{aligned} \begin{gathered} Pr(S_i = 1) = \texttt {Logit}^{-1}\bigg (\beta _1Gender_i + \beta _2Bronze_i + \beta _3Silver_i + \beta _4Gold_i + \beta _5Grand Prix_{i} + \\ \beta _6Competitions_i + \beta _7Prestige_{ki} + \epsilon _i \bigg ) \end{gathered} \end{aligned}$$

**Figure 2:**
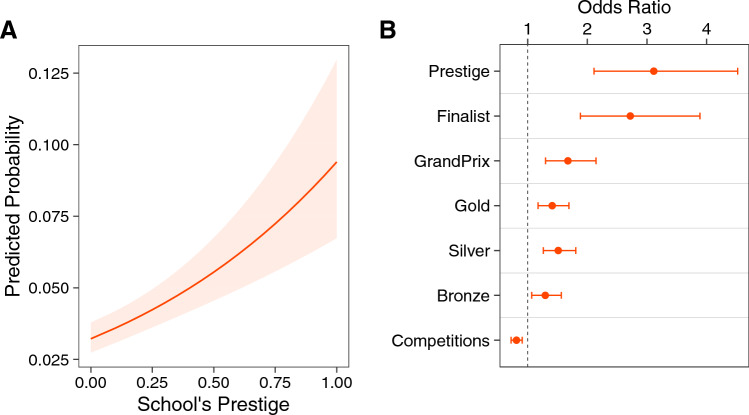
Probability of job success in ballet. Success is defined in a binary fashion, where $P(S=1)$ if the student obtained a job placement in a ballet company, and $P(S=0)$ otherwise. (**A**) Demonstrates a significant positive effect on the predicted probability of a job placement with the increase of school’s prestige. (**B**) Shows exponentiated odds ratio with corresponding 95% confidence intervals for the effect on job placement of each additional unit of competition outcomes and institutional prestige. Baseline in 1 indicates no effect. We see that the Grand Prix has the largest effect by type of awards, while long competition trajectories can be detrimental for a job placement, and being a finalist is comparable to be affiliated to ahighly prestigious school. Model coefficients reported in Table 1.

We observe a strong positive effect of prestige on job placement (Model 1 in Table 1). Moreover, our analysis reveals a significant increase in the probability of job placement along with schools’ increasing prestige (Fig. 2A). For example, consider two comparable ballet dancers, Lauren and Juliet, who both won one gold medal after two competition appearances, but who attend schools of differing prestige: Lauren attends a school with prestige 0.87 while Juliet’s school has a prestige of 0.09. Our logit model predicts that despite their identical competition performance, Lauren’s probability of a job placement is 2.25 times higher than for Juliet.

Next, we test for the potential effect of advancing to the competition finals on job placement by adding a dummy variable for being a finalist ($F_i = 1$) or not ($F_i = 0$). In this second model, we observe a strong effect on the probability of a successful job placement (Model 2 in Table 1, AUC = 0.7327), which is comparable to the effect of being affiliated to the most prestigious schools (see Fig. 2B). This comparison suggests that being a finalist can greatly enhance the career prospects of talented students who attend less prestigious schools, and highlights the significant impact of high performance on a job placement.

Our logistic model can also reveal more detailed effects of medals and competitions on job placement. Intuitively, examining medals by type (model 2 in Table 1, Fig. 2B), the odds ratio increases with medal importance: winning a bronze medal increases the odds of a job placement by 30% compared to a no medal baseline, while one additional gold or silver medal increases those odds by about 50%. The greatest impact of awards on a student’s odds of attaining a job placement comes from winning the Grand Prix, a special recognition given on the subjective appreciation of the jury, increasing the odds of a successful job placement by 67%. This suggests that the recognition of a dancer by the jury is highly aligned with the value system adopted by ballet companies, much more than winning multiple competition medals awarded on a technical scoring system.

Our analysis also highlights an unexpected finding: a long competition career may negatively impact job placement. On average, students participate in two semi-final competitions regardless of their job placement outcome. However, our analysis shows that each additional semi-final competition decreases the chances of a job placement by 18% (model 2, Fig. 2B), which indicates that students who participate in multiple competitions may not improve their chances to be recruited.

Overall, we find that school prestige has the largest effect in determining job placement, with the odds increasing by over 200% for students who attend the most prestigious schools, and this effect is robust across all models (see more model variations in SI, Section S3.2). Yet, while our results also emphasize the importance of high performance as a key factor for career success, these models are unable to disentangle potential interactions between performance and prestige.

**Table 1:** Model coefficients for the logistic models predicting successful job placement.

	Dependent variable = Success $S_i$	
	(1)	(2)
Prestige	$$1.201^{***}$$	$$1.136^{***}$$
	(0.190)	(0.193)
Grand Prix	$$0.809^{***}$$	$$0.515^{***}$$
	(0.113)	(0.127)
Gold	$$0.45^{***}$$	$$0.344^{***}$$
	(0.091)	(0.093)
Silver	$$0.45^{***}$$	$$0.413^{**}$$
	(0.091)	(0.091)
Bronze	$$0.276^{**}$$	$$0.258^{**}$$
	(0.096)	(0.097)
Finalist $$F_i$$		$$1.0^{***}$$
		(0.184)
Competitions	$$-0.238^{***}$$	$$-0.208^{***}$$
	(0.058)	(0.058)
Gender: male	$$0.754^{***}$$	$$0.756^{***}$$
	(0.121)	(0.121)
Constant	$$-3.135^{***}$$	$$-3.193^{***}$$
	(0.111)	(0.112)
*Note:*	$$^{*}p<0.1$$; $$^{**}p<0.05$$; $$^{***}p<0.01$$	
Observations	6393	6393
McFadden pseudo $R^2$	0.085	0.095
AIC	2640.3	2615.3
AUC	0.7327	0.7374

Model coefficients labeled by *p*-value. Standard errors in parentheses.

To further elucidate the role of school prestige on individual job placement, we conduct an experiment in which we match students who have identical medal and competition counts, but who differ on their school’s prestige. Here, the YAGP medal counts function as a proxy for dancer ability, empowering us to measure the influence of prestige beyond performance.

We consider a binary treatment status denoted as $Y_i = 1$ for the students affiliated to a prestigious school, and $Y_i = 0$ for students who attended a less prestigious school. The subset of prestigious schools comprises the top 5% of the network-based ranking of prestige. Under this criteria, we assign 93 top schools as $Y = 1$, resulting in 2301 treated students and 3780 controls (132 subclasses). We match the observations with the exact matching method using MatchIt^53^. The exact matching is performed over the quantified variables of individual achievement, including: (1) total number of each competition medal (gold, silver, and bronze medals); and (2) the total number of competitions, both listed only in the semi-finals. Finally, the matching model can be described as:

3 $$\begin{aligned} E(S | Y=1, X) - E(S | Y=0, X) = \frac{1}{N}\sum _{i=1}^{N}(S_i - S_{j(i)}) \end{aligned}$$

where *S* is the job placement outcome, *Y* is the treatment indicator, *X* contains the vector of covariates used for exact matching, *N* is the number of subclasses formed in the matching process, and *j*(*i*) is the number of controls used to match the treated observation (*i*). We compute the matching method specifying the Average Treatment Effect as the estimand and heteroscedastic-consistent standard errors based on subclasses^54^.

We observe that, by comparing equally skilled dancers, i.e. students who have exactly the same competition outcomes, there is a significant increase of 65% in the odds of obtaining a job placement ($p < 0.001$) for those who attended a prestigious school. This smaller effect size compared with the logistic regression model 2 (shown in Table 1 and Fig. 2), where we observe an effect of 200% on the odds of obtaining a job placement, occurs because the matching experiment accounts for potential interactions between school prestige and medal counts by comparing equally skilled individuals within subclasses (dancers that obtained the same number and type of competition medals). Our results show that the effect of social prestige is reduced from a 200% increase to a 65% increase after matching based on performance. This suggests that we successfully decreased the bias in our estimation due to observable confounders. These findings suggests that even though dancers can obtain a similar number and type of competition medals, an indicator of similar ability and performance, their affiliations play a crucial role in their careers, which ultimately influences their professional positioning in a ballet company.

**Figure 3:**
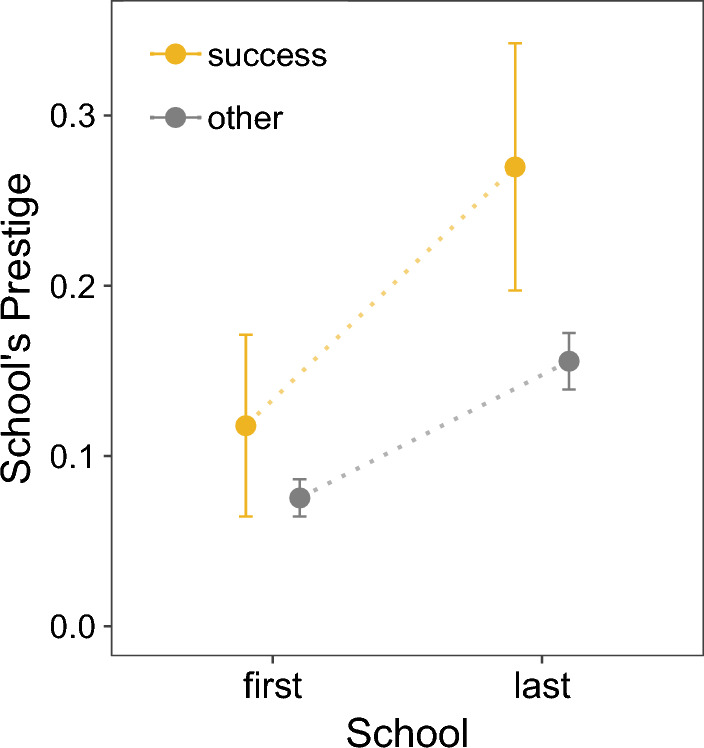
Change in school’s prestige for transfer students. The change in school’s prestige (mean with 95% confidence intervals) from students who attend more than one ballet school during their participation in the competition. We observe that students move to more prestigious schools, and students with a successful job placement (labeled as ‘success’, in yellow) move to even more prestigious schools compared to those without a job placement.

Given the positive impact of school prestige on job placement, we further investigate if students who change schools move to a more prestigious school. While 85% of all participants only reported one school affiliation, the remaining 15% (932 students) attended from two up to five schools. From the students who changed schools, 85 students (10%) obtained a successful job placement. To capture the difference in schools’ prestige, we first measure the change in prestige from students’ first and last schools. Then, pairing each student by their first and last school, we find a difference of 0.086 in the schools’ prestige between the last and first school (last: mean = 0.165, sd = 0.258, first: mean = 0.079, sd = 0.17), indicating that students tend to move to a more prestigious school ($t=8.49, p<0.001$). We further examine the school change by comparing the first and last schools by students’ job placement outcome. We measure the difference in schools’ prestige with a two-way ANOVA test revealing that the change in schools (first/last) shows an increase in school’s prestige in general ($F = 73.725, df = 1, 1860, p < 0.001$). Moreover, we observe a larger increase in school’s prestige for students who obtained a successful job placement ($F = 19.907, df = 1, 1860, p = p < 0.001$). The interaction effect was also significant ($F = 4.16, df = 1, 1860, p = 0.0414$). The difference in the change of schools’ prestige by each group can be seen in Fig. 3.

Finally, we investigate the impact of awards on the careers of students who change schools. For each of the 795 students that only changed a school once, we control for the highest award received while at the pre-change school. We then measure the difference in school prestige for each student as $\Delta Prestige = Prestige_{School_2} - Prestige_{School_1}$; thus a $\Delta Prestige > 0$ reflects an increase in affiliation prestige, $\Delta Prestige < 0$ a decrease in affiliation prestige, and $\Delta Prestige = 0$ is no change. Using a One-Way ANOVA test we observe no effect of the highest award of School 1 on the prestige of School 2 ($F(4, 790) = 0.565$, $p = 0.687$). Because $\Delta Prestige$ is not normally distributed (Shapiro-Wilk test, $p < 0.001$), we confirmed this analysis with a Kruskal-Wallis test and found no statistical effect ($\chi ^2(4) = 4.5836, p = 0.3327$). These results suggest no relationship between previous awards obtained in School 1 and the change in school’s prestige of School 2, and may be a indicator that the change of students to more prestigious schools could be driven by other mechanisms, such as self-selection or peer effects. The distribution of $\Delta Prestige$ across awards levels obtained in School 1 are shown in SI Fig. S7.

Overall, our results emphasize the importance of social prestige for a successful job placement in ballet and show that students may have access to more prestigious institutions over time.

## Summary and discussions

In summary, our research highlights the usefulness of the *science of science* methods as an efficient tool to quantify career patterns in creative professions that were not possible to elucidate before. The joint use of *science of science* and network science allowed the identification of the leading ballet academies in the US. This contributes to expand the general understanding of the arts academic system in the US and its relationship with reputation and prestige^55^. Moreover, our work also demonstrates that features of artistic careers can be quantified, and emphasizes previous efforts of researchers investigating the different factors driving the evolution of the arts in an objective fashion^49^.

Our work unveils the importance of both individual competition performance and schools’ prestige as predictors of successful job placements in ballet. By systematically measuring schools’ prestige through network analysis, we demonstrate that social prestige is predictive of higher jury’s recognition of students, competition advancement, and better career prospects. As a whole, we show that the social network remains essential to shape success in ballet’s modern era, and illustrate the potential of data-driven methods to objectively analyze these effects in performing arts.

The pursuit of a successful career in ballet often requires young dancers to give up their childhood, as demanding training regimes are essential to attain the level of athleticism and motor control necessary to execute complex, yet artistic, movements and sequences. Despite the rigorous physical preparation, the history of ballet suggests that the selection and advancement of dancers is influenced by more than just performance ability, and is strongly shaped by the prestige of social and professional connections. In the modern era, dancers can leverage this principle by affiliating with prestigious academies that provide access to the network of experts who play a critical role in identifying and promoting rising stars.

Through our examination of the network of ballet academies in the United States, we provide a network-based ranking of these academies, and reveal the hierarchical social stratification of prestige within the ballet academic environment. This validated network-based measure of prestige in ballet complements similar measures of prestige in academic careers^12,44,45^, visual arts^21^, and the movie and music industry^15^. For instance, being central in a collaboration network of performing artists correlates with the better allocation of resources and more impact of creative performance^20,22,23^. In the field of visual arts, the top 20% more prestigious art galleries and museums predict a 58.6% higher individual reputation, which also relates to higher sales rates and a longer career^21^; while our results show an effect of 65% to secure a job placement in a ballet company from being affiliated to the top 5% more prestigious schools. A similar effect of early career recognition and institutional prestige has been reported in academia for career development and scientific impact^56,57^. Interestingly, our study emphasizes the short-term effect of pre-professional competition awards and a negative effect of multiple competitions on the successful job placement of dancers in their early career. In contrast, similar analysis on academic careers suggests a cumulative advantage of early achievement for future rewards and recognition^58,59^. However, our analysis only captures the short-term effect of awards and social prestige on job placement, and may be subject to a selection bias in which successful dancers are not incentivized to compete in additional competitions. Future research can help reveal dancers’ career dynamics and the cumulative advantages of early social recognition for promotion and role allocations within the context of company turnover rate and market demands. Overall, the nuances of network effects across creative domains raises the question of the role that social complexity plays for career success, also considering other factors such as the nature of connections formed over time^60^ or embedded formal and informal norms^61^. Thus, additional comprehensive longitudinal data on dancers’ careers and company structures could help further investigate the long-term effects of dancers’ achievements and institutional prestige for career development and longevity.

The ballet industry is renowned for its limited job opportunities and high competitiveness. Our research shows that ballet companies often exhibit selection biases based on the dancer’s affiliations. This is a common issue in competitive settings, where evaluators find it challenging to differentiate between similarly talented candidates^38,62^. In such cases, evaluators tend to make their selections based on social cues, such as the prestige of affiliation, and personal biases. Thus, the relationship between affiliation prestige and dance ability is complex, and may involve reverse causality. On one hand, a prestigious institution may attract high-quality dancers by means of specific recruitment criteria, which can in turn reinforce dancers’ prestige and provide access to better training opportunities. On the other hand, a high-quality dancer may also enhance the prestige of their affiliation as a result of their talent. To counteract selection biases, an adequate implementation of blind auditions could increase fairness in the selection of talented candidates from less prestigious institutions^63^.

Several limitations of our research should be taken into consideration. First of all, our data is limited to the YAGP competition outcomes and presents a school-level metric of social prestige to measure the impact on successful hiring at an individual level. In addition, metrics of individual performance may as well imply endogeneity imposed by high achievement and job placement. While our matching experiment controls for equal individual performance, the experiment assumes that the treatment of being in a prestigious school does not depend on the treatment assignment of other individuals. However, ballet schools may be able to accommodate only a fixed number of students, and elite schools may restrict enrollment to maintain exclusivity or prestige, which may limit the treatment assignment of being in a prestigious school. Thus, the assumption of no interference in treatment assignment may not hold in the context of ballet schools, and may require specialized statistical techniques to address this issue in further research. Moreover, our analysis does not account for omitted variables and unobserved factors such as certain standards of beauty, behavior, technique style and repertoire, years of experience in ballet competitions, personal choices, market demands, and other attributes that ballet companies may consider in their hiring process. In a similar fashion, the data analyzed here does not hold information about the judge pool for the competition and the hiring teams of ballet companies, which may influence the allocation of awards and job placements.

The nature of the data also limits our ability to measure peer effects on competition or career achievement^64,65^ derived from the competition setting. Yet given that our measure of prestige may be confounded by peer effects from the competition setting, thus it may capture these potential effects due to its aggregated nature. However, a deeper investigation on this matter would help understand how dancer’s decision is shaped by school changes, persistence in the competition, or pursuing a professional career in relation to their local network^66^ and the behaviors of their peers. Similarly, we were unable to capture the influence of other individual dancers’ rewards, including scholarships for summer or yearly training in prestigious academies. Although our findings provide insights into the potential benefits of affiliation with a prestigious school for career success in the ballet industry, it only reflects the hiring process for YAGP participants and may not be representative of the entire population of young ballet dancers. Also, there are several other U.S.-based and international competition substitutes to the YAGP, like the World Ballet Competition or the Prix de Laussane, which could similarly influence student career outcomes.

While our measure of success currently focuses on job placements as company dancers, it is important to recognize that a successful career in ballet can encompass a variety of roles, including teaching, choreography, and administrative duties. Therefore, there is a need for more comprehensive data to investigate the career paths of ballet dancers, from pre-professional to professional levels, allowing our definition of success to include diverse career paths. With richer data, we can further investigate the complexity of human capital and the job market^67^ within creative professions. This exploration would allow us to map socioeconomic variables shaping the structure and evolution of dancers’ careers, while also exploring the interplay between factors like school choice, institutional prestige, skill hierarchy, and career success.

Lastly, we hope that this work raises the attention of how important is school choice for the dancers’ future and how interdisciplinary research contributes to the understanding of human creativity at a social level, which can ultimately inform about the underlying mechanisms driving the evolution of arts and our cultural heritage.

## Methods

### Dataset

We use publicly available data from the YAGP online platform^68^. The data used in this study was collected from the YAGP Winners’ report (https://yagp.org/winners/) and the alumni success stories (https://yagp.org/alumni-success-stories/). We employed the BeautifulSoup Python library for web scraping^69^, adhering to ethical guidelines and terms of service of the platform. To protect the privacy interests of the dancers, their names are anonymized by converting them into sequenced numbers, which facilitates their handling, and the identity key was stored separately. Our data collection and research methods were approved on January 18th, 2023, by the Institutional Research Ethics Committee of Universidad del Desarrollo, in Chile. In addition, the use of the public data resources was authorized by Larissa Savaliev, director of the YAGP.

The data contains the competition results of 10,686 students and 2402 schools participating from 2000 to 2021. We subset the data to only include the 6363 students listed from competition venues within the United States (namely, Youth America Grand Prix) for a robust representation of the competition system. This selection of students comprises those in the professional age range, which filters out students from ‘Pre-Competitive Age Division’ after 2014, and ‘Junior Age Division’ after 2019. All students from the ‘Senior Age Division’ are considered for this analysis. In total, our student population is 7% Pre-Competitive Age Division, 28% Junior Age Division, and 65% Senior Age Division.

To disambiguate the students and schools, we first checked for misspellings and punctuation. We then performed an exact name matching that leverages middle names and/or initials to distinguish identity, for both students and schools’ names. The final data contains 6475 participants, from which 6,393 students are affiliated to any of the 1603 ballet schools found in the data. We infer the gender of students using the gender package for R, a method of binary gender inference (Woman, Man) that matches names with their gender as found in the package standardized databases (ssa, ipums, napp, and demo)^70,71^. This method’s estimation uses the probability of finding a gender assigned to a given name; when the probability is larger than or equal to 0.7, then the gender is assigned to the name tested. Only 0.008% of students’ gender was not identified and were removed from the dataset. Its important to emphasize that the inferred gender does not refer to the sex or self-assigned gender of dancers, but serves as an estimate of the social construction of gender. Also, students’ reported gender can be confirmed in the YAGP website records if necessary. Overall, women represent 83% of the total population, representing a self-selection gender bias embedded in the competition system.

### Measure of social prestige

The network of ballet schools is represented as $G = (K, V)$, where *K* is the set of schools (nodes, *k*) and *V* the set of connections between schools (edges, *v*). Hence, there is an edge (*v*) between two schools $k_1, k_2 \in K$ so that $v(k_1, k_2) \in V$ if their affiliated students are listed as top student in the same competition venue and year.

From this network, we compute the unweighted betweenness centrality, following Eq. (1)^51^. Betweenness centrality is then normalized with the Min-Max scaling method to have a linear range between [0, 1], where $B_k = 1$ corresponds to the most central school. We then order the schools by their normalized centrality and create a ranking list using a dense rank function, which generates rank ties for observations with the same centrality values. The rank *r* of a school *k* by its centrality ($r = \{1, 2, \ldots , K \}$) is assigned in an ascending fashion, so that $r_k = 1$ is the largest centrality value, and $r_k=945$ will have the lowest centrality in the set of schools *K*. We provide a detailed explanation of the validation methods regarding the ranking of social prestige in SI, Section S2.3, where we compare our ranking with third-party lists of prestigious schools selected by dance experts.

### Supplementary Information

Supplementary Information 1.

## Data Availability

The datasets generated and/or analyzed during the current study are available in the Zenodo repository, link here.
