# Supplementary material for: Quantifying hierarchy and prestige in US ballet academies as social predictors of career success
Source: Sci Rep. 2023 Oct 30;13:18594. doi: 10.1038/s41598-023-44563-z (PMC10616162; doi:10.1038/s41598-023-44563-z)
Supplement: Supplementary file 1 — Supplementary Information 1. [file 41598_2023_44563_MOESM1_ESM.pdf]

Supplementary Information for

Quantifying Hierarchy and Prestige in US Ballet Academies as Social Predictors of Career Success

Yessica Herrera-Guzmán, Alexander J. Gates, Cristian Candia, Albert-László Barabási*

*Corresponding Author. Email: alb@neu.edu

This file includes:

Supplementary Text

Figures S1 to S7

Tables S1 and S2

References

Contents

S1 Youth America Grand Prix 3

S2 Ballet Schools 3

S2.1 Distribution of Ballet Schools across the U.S. 4

S2.2 Schools' achievement 4

S2.3 Ranking of Ballet Schools 6

S3 YAGP Alumni 11

S3.1 Students' achievement 11

S3.2 Students' success 14

References 17

S1 Youth America Grand Prix

The Youth America Grand Prix (YAGP) is a highly influential ballet competition that aims to discover and reward young dancers. This paid event provides education and job opportunities to dance students in pre-professional training, and is seen as an effective way to promote dancers on the path to successful careers. The competition is held in two stages, with multiple regional semi-finals and one final competition each year. Participants are divided into age categories: Pre-Competitive (9-11 years old), Junior (12-14 years old), and Senior (15-19 years old; 20 years old after the COVID-19 pandemic in 2020).

The competition recognizes dancers with two types of awards. The first are the competition medals: gold, silver, and bronze, which are given based on aggregate scores across various judges and dance categories. The hierarchical top three positioning is based on a 1-100 scoring system where the competition jury evaluates technical and artistic elements of performance. The medals (gold, silver, bronze) are given to the highest scores in each competition group formed by Division and Category in each venue. The second is the Grand Prix, which is awarded based on the subjective appreciation of exceptional performance by a committee of judges without explicit criteria. The Grand Prix is not always awarded, thus is considered the highest distinction of the competition.

Both awards can be given during the semi-finals or finals; the medals may reflect ties, while the Grand Prix is only awarded to one student per division, or may not be given at all. The YAGP publicly reports the medal and Grand Prix winners, and overall top students (up to top 12) by year and location. As many YAGP competitors are awarded scholarships and professional contracts in ballet companies, the YAGP also reports on the successes of its alumni, including their pre-professional affiliations and current job placements.

S2 Ballet Schools

We define a 'ballet school' as any organization that offers ballet training. In the US, the structure of ballet schools vary in their organization. There are both university and non-university programs. For instance, the Higher Education Arts Data Services 2020-2021 report lists 76 universities affiliated to the National Association of Schools of Dance. For the context of this research, we must consider that most dancers competing at YAGP would engage in pre-professional ballet training in non-university institutions because of the competitive age divisions, ranging from 10 to 19 years old.

S2.1 Distribution of Ballet Schools across the U.S.

We explore the geographic location of schools to map the distribution of ballet academies across the US. To do this, we perform a Google search of all schools' addresses to find their georeference. Simultaneously, we confirm the formation of communities in the network of ballet schools using the Louvain algorithm [1], as this helps understand whether schools tend to compete in clusters. We find that the communities detected with the algorithm and their geographical regions strongly overlap. We show the resulting network in Figure S1 with colors assigned based on the community detected and node's position based on actual US regions, as: Northeast, East, Midwest, South, and West.

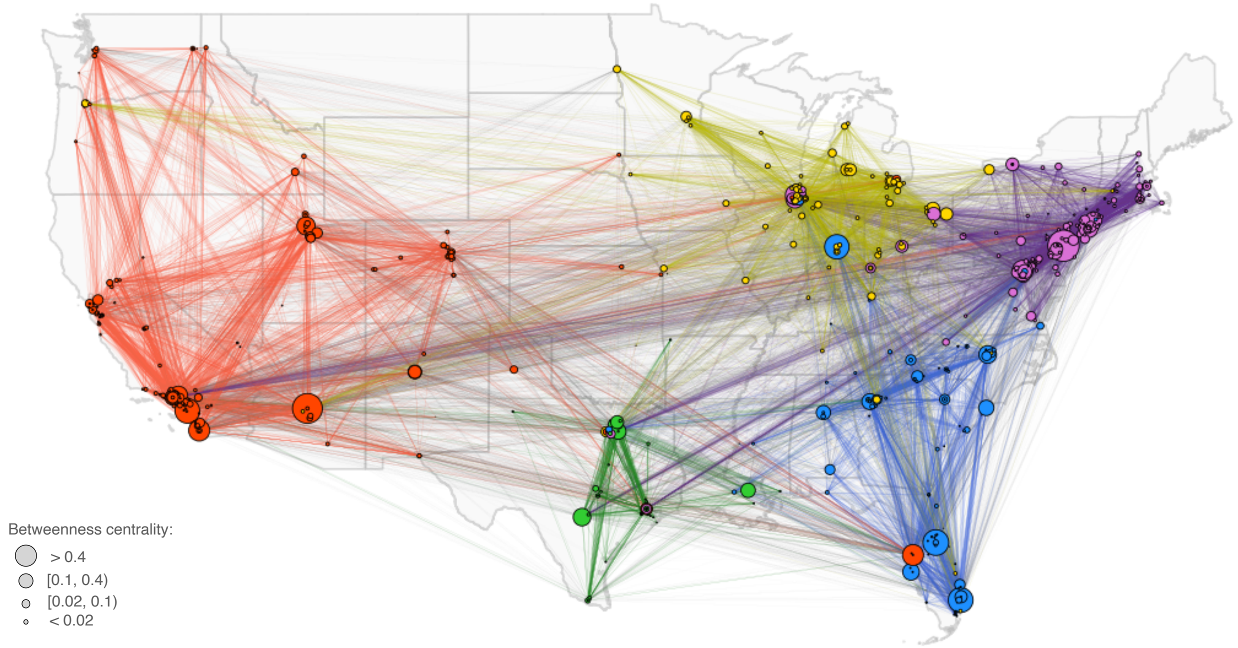

Figure S1: **Network of ballet schools in the US.** Ballet schools are nodes, and two schools are linked by winning in the co-competition of the YAGP. Node size by schools' betweenness centrality, B_k. Schools' location corresponds to their actual geographical location. Nodes are colored by detected communities: Northeast (purple), East (blue), West (orange), South (green), and Midwest (yellow). Edge color/gray for intra/inter community links.

S2.2 Schools' achievement

Achievement, measured by the number of awards received per school, is an important factor influencing prestige because it can capture the level of social recognition that one institution has for its outstanding performance. Here, we explore schools' achievement in the YAGP competition and the existing patterns in the distribution of awards, to test the relationship with network

centrality. This analysis provides insights on whether social prestige is rooted in achievement, or it is also related to the richness of professional connections.

From the YAGP's structured data, we quantify schools' achievement as a proxy derived from the number of awards received during their participation in the competition. We compute the following metrics:

i. *School's total number of awards, A_k*: the sum of awards a from the total competitions J (semi-finals and finals), that each student i from the set of students I obtains per competition j when they are affiliated to school k. This can be noted as $A_k = \sum_{i=1}^{I} \sum_{j=1}^{J} A_{ij}\theta_{ijk}$, where A_{ij} is the individual sum of awards per competition, $\theta_{ijk} = 1$ is given if the student i is affiliated to school k for the competition j, and $\theta_{ijk} = 0$ otherwise.

ii. *Ratio of awards per school, R_k*: the ratio of obtained awards derived from the number of competitions where affiliated students ranked as top 12. The ratio of awards for schools is $R_k = \frac{A_k}{T_k}$ where T_k is the total top students affiliated to school k, given by $T_k = \sum_{i=1}^{I} \theta_{ik}$ for $\theta_{ik} = 1$ if a student is affiliated to k, and $\theta_{ik} = 0$ otherwise; then R_k gives the number of awards per student affiliated to each school. Here, $R = 0$ indicates that no awards were obtained, $R = (0, 1)$ indicates less than one award per competition, $R = 1$ gives an even relation between the number of awards and competition, and $R > 1$ for those with more than one award per competition in top 12. We label each group as 'no medals', 'under-achiever', 'break-even', and 'high-achiever', respectively for each ratio group mentioned above.

Figure S2A shows the total number of awards per school, from which we observe a fat-tail distribution, indicating that most schools who have won awards have obtained only one or two awards, while a few schools collected more than 100 awards. To better understand the relationship between the total number of awards (A_k) and the total number of students (T_k) per school k, we compute the ratio of awards as $R_k = A_k/T_k$. From this ratio, four groups of schools are observed (see distribution of in Figure S2B). We find that about 75% of schools have a low ratio of awards. For instance, 692 schools (43.2% of all) did not receive any awards ($R_k = 0$), even though they had top students listed by the YAGP; and 519 schools (32.4%) are 'under-achievers', with less than one award per top student ($R_k = (0, 1)$, in blue). Conversely, only 227 schools (14.1%) have a 'break-even' ratio ($R_k = 1$, in yellow), indicating one award per top student; and only 10.3% of schools (165 schools) are 'high-achievers' ($R_k > 1$, in red), meaning that their students obtain more than one award. Noteworthy, the 'high-achiever' group ranges from very low to a higher number of students, indicating that a high ratio of awards is independent of the frequency of schools' participation in the competition.

Next, we examine the relationship between schools' ratio of awards and betweenness centrality. In Figure S2C, we observe that most schools are located in the lower quartile of betweeenness

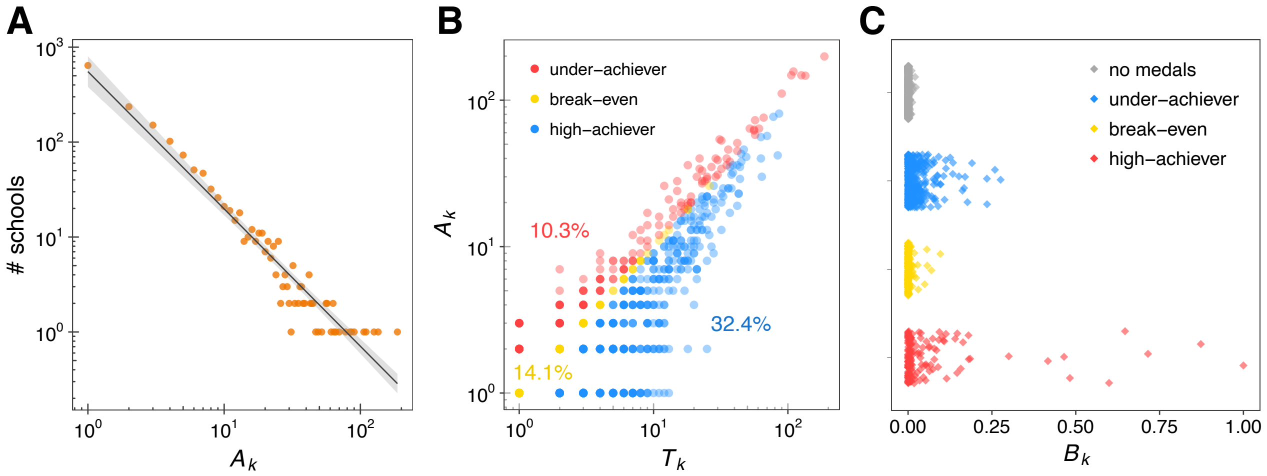

Figure S2: **Awards and centrality of ballet schools.** **A** shows the fat-tailed distribution of total awards per school, A_k, indicating that from those schools obtaining awards, most of the have only one, and only a few have more than more than 100 awards. **B** shows the log distribution of awards A_k respect to the number of students T_k per school k, i.e. ratio of awards, R_k. Color indicates the corresponding group by ratio of awards and their percentage (plot not displaying schools with 'no medals', $A_k = 0$). Only 56.8% of schools obtained at least one award, and the total number awards increases proportionally to the number of students. However, the ratio of 'high-achievers' is independent from their number of top students. **C** shows the relationship of betweenness centrality, B_k, with each group by ratio of awards. Schools with no medals (in gray) have the lowest centrality value, while only highly central schools are in the 'high-achievers' group.

centrality, B_k. Schools with no medals (in gray) have the lowest centrality values, closer to zero. On the contrary, only highly central schools are in the 'high-achievers' group, yet this group also includes low centrality schools. Moreover, 'under-achiever' schools have a wider distribution across the lower quartile, indicating that these schools can have higher centrality than the other groups.

Taken together, these findings help contextualize the existing relationship between achievement and its variation by schools' network position.

S2.3 Ranking of Ballet Schools

To rank schools by their social prestige, we first evaluate different network centralities and schools' achievement to test what measure captures prestige more accurately. Then, we validate our ranking method by comparing our metrics with an external selection of prestigious schools from dance experts. The network centralities are listed in Table S1 and were selected based on the reported association of network position and social prestige [2]. Achievement is measured by the number of awards received per school (see SI S3.1), an important factor influencing prestige because it can capture the level of social recognition that one institution has for its outstanding performance [3, 4]. To control the effect of the number of awards (A_k) by the number of students

(T_k, a proxy of schools' size), we compute the ratio of awards as $R_k = A_k/T_k$ and include it as a metric of achievement.

Table S1: Centrality metrics to assess the prestige of ballet schools. Formula in general notation as described in the specified citation.

Network measure	Description	Formula
**Betweenness**	how much a node connects two other nodes	$B_i = \sum_{s,t} \frac{n^i_{s,t}}{n_{s,t}}$ [5]
**Closeness**	a node's proximity to other nodes	$Ci = \frac{n-1}{\sum_{v=1}^{n-1} d(v,u)}$ [6]
**Degree**	a node's connectivity	$D_i = \frac{k_i}{N-1}$ [7]
**Eigenvector**	a node's influence	$E_i = \frac{1}{\lambda} \sum_k a_{k,i} E_k$ [8]

Separately, we collect an external selection of prestigious schools by ballet experts from different sources. First, *Dance Magazine*, a leading multimedia platform in the dance world, that also partners with other dance sources in multiple publications (e.g. Pointe, Dance Spirit, and Dance Teacher). We use their selection of top pre-professional academies for the academic year 2022-2023 [9]. Separately, we collect the list of top ballet schools in the US and the 2023 Summer Intensive Guide from the blog *A Ballet Education*, one of the most reliable online ballet experts for teachers, students, ballet professionals, and ballet lovers [10]. In total, the list of top schools formed from these sources, contains 60 schools' names, shown in Figure S3.

We implement a classification model using a binary system for the list of Top Ballet Schools (1:top school, 0:no top school) to test the schools' centrality and achievement measures' accuracy to capture social prestige. Considering each metric in separate models, we measure the area under the cure (AUC) of the classification model (Receiver Operating Characteristic Curve, ROC) using the `pROC` package for R [11]. The AUC is a score between 0 and 1, where a value closer to 1 indicates a probability of correct classification, while a score lower than 0.5 indicates that the model performs no better than random guessing. AUC and ROC for each centrality are shown in Figure S4. The AUC=0.75 suggests that betweenness centrality (Fig. S4A) is the most accurate centrality measure capturing social prestige, respect to the other centrality metrics (Fig. S4B-D). On the other hand, when we explore the ratio of awards (number of total awards controlled by school size) we observe the lowest accuracy in predicting schools' prestige (Fig. S4F), with an AUC=0.71.

Further, we order the schools by their betweenness centrality and create a ranking list. In this rank, a school k with $r_k = 1$ has the largest centrality value, i.e. is more central or prestigious, and the largest rank value (e.g. $r_k = 945$) has the lowest centrality in the set of schools K. Based on the number of observations per school, we select the upper 5% of schools from the

1.The Rock School for Dance Education, PA
2.Master Ballet Academy, AZ
3.Indiana Ballet Conservatory Inc, IN
4.Southland Ballet Academy, CA
5.Orlando Ballet School, FL
6.The Art of Classical Ballet, FL
7.Ballet West Academy, UT
8.Ellison Ballet, NY
9.Cary Ballet Conservatory, NC
10.Metropolitan Ballet Academy & Company, PA
11.Anaheim Ballet Academy, CA
12.Colorado Ballet Academy, CO
13.The Sarasota Cuban Ballet School Inc, FL
14.Maryland Youth Ballet, MD
15.Elite Classical Coaching, TX
16.Joffrey Ballet Academy, NY
17.Sarasota Ballet School, FL
18.Joffrey Academy of Dance, IL
19.Cincinnati Ballet Otto M. Budig Academy, OH
20.The Ballet Clinic, AZ
21.Koltun Ballet Boston, MA
22.Harid Conservatory of Music Inc, FL
23.Texas Ballet Theater School, TX
24.Timothy M Draper Center for Dance Education Inc, NY
25.Central Pennsylvania Youth Ballet, PA
26.Kansas City Ballet School, MO
27.Vitacca Vocational School for Dance, TX
28.City Ballet San Francisco, CA
29.BalletCNJ, NJ
30.San Francisco Ballet School, CA
31.Houston Ballet Academy, TX
32.Milwaukee Ballet, WI
33.A&A Ballet, IL
34.Boston Ballet School, MA
35.BalletMet Dance Academy, OH
36.Interlochen Center for the Arts, MI
37.Ballet Conservatory at Skyra, FL
38.Nashville Ballet, TN
39.West Met Classical Training, MN
40.National Ballet Academy Denver, CO
41.Nutmeg Conservatory, CT
42.Pittsburgh Ballet Theatre, PA
43.Pennsylvania Academy of Ballet, PA
44.The School of American Ballet, NY
45.School of Ballet 5:8, IL
46.Dmitri Roudnev Ballet School, IL
47.Oregon Ballet Academy, OR
48.Neglia Ballet, NY
49.Tulsa Ballet Center for Dance Education, OK
50.Sacramento Ballet, CA
51.Alonzo King LINES Ballet School, CA
52.Saint Louis Ballet School, MO
53.Oklahoma City Ballet Yvonne Chouteau School, OK
54.Philadelphia Ballet, PA
55.Ballet Austin Academy, TX
56.Jacqueline Kennedy Onassis School at ABT, NY
57.Miami City Ballet School, FL
58.State Street Ballet, CA
59.The Portland Ballet, OR
60.Princeton Ballet School, NJ

Figure S3: **List of top schools.** Selection of top schools by US experts from *Dance Magazine* and *A Ballet Education*. Schools listed in no particular order.

network-based ranking. The list of schools is shown in Figure S5. We use this selection for the further treatment effect of being in a top school on job placement, notated as $Y_i = 1$ for students affiliated to a prestigious school, and $Y_i = 0$ for students who attended a less prestigious school.

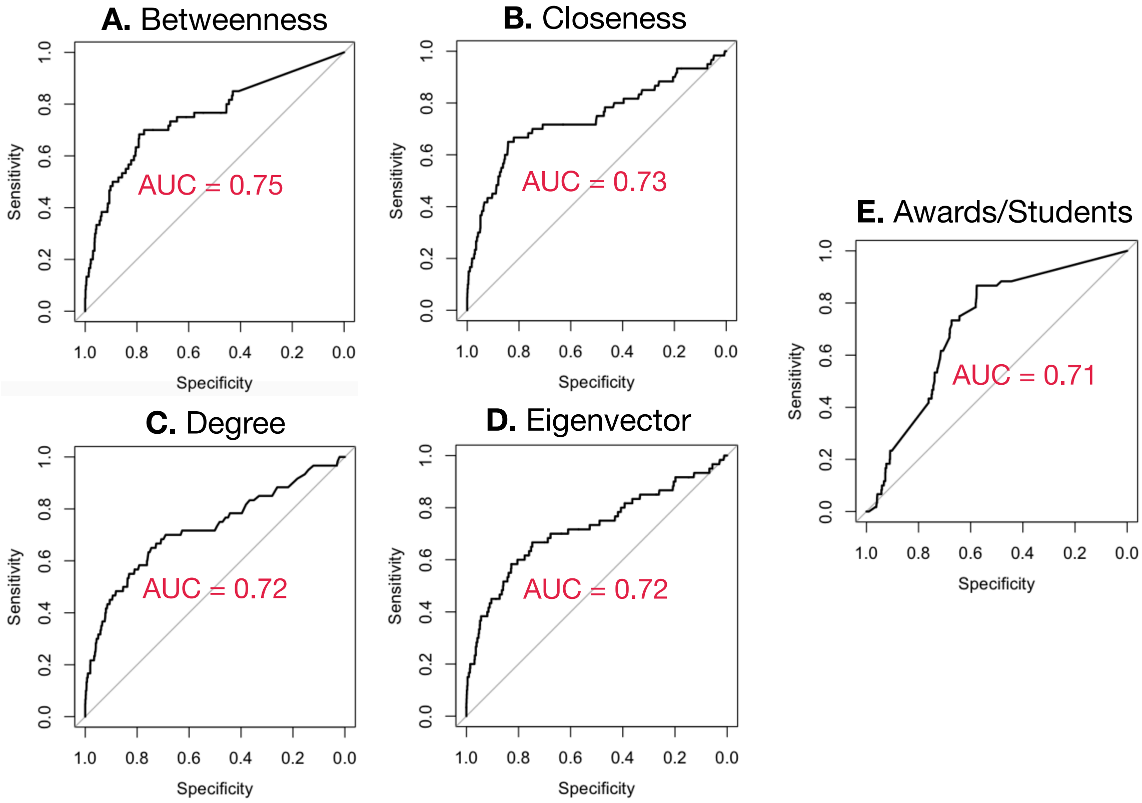

Figure S4: **AUC for network centrality and ratio of awards.** Panel **A** shows that betweenness centrality is the best indicator of prestige with the largest AUC=0.75 among all centralities in panels **B-D** and schools' achievement, in panel **E**.

1.The Rock School for Dance Education, PA
2.Master Ballet Academy, AZ,
3.Indiana Ballet Conservatory Inc, IN
4.Dmitri Kulev Classical Ballet Academy, CA
5.The Kirov Academy of Washington, DC
6.Southland Ballet Academy, CA
7.Orlando Ballet School, FL
8.Marat Daukayev Ballet Theatre Inc, CA
9.Faubourg Theatre Inc, IL
10.The Art of Classical Ballet, FL
11.Danceology Academy of Classical Ballet, CA
12.Next Generation Ballet, FL
13.University of North Carolina School of the Arts, NC
14.Ballet West Academy, UT
15.Pacific Coast Academy of Dance, CA
16.Ellison Ballet, NY
17.The Dallas Conservatory, TX
18.The Academy of Ballet Arts Inc
19.The Washington Ballet School, DC
20.The Academy of Dance Arts, IL
21.International Ballet School, CO
22.CityDance School & Conservatory, MD
23.Dance Center of San Antonio, TX
24.V&T Classical Ballet & Dance Academy, CA
25.The Rock Center for Dance, NV
26.International City School of Ballet, GA
27.Alwin School of the Dance, NM
28.Ballet Conservatory, TX
29.Cary Ballet Conservatory, NC
30.Westlake School for the Performing Arts, CA
31.Metropolitan Ballet Academy & Company, PA
32.Akhmedova Ballet Academy, MD
33.Greenwich Ballet Academy Inc, CT
34.Jacqueline's School of Ballet, UT
35.Classical Ballet Academy, CA
36.Odasz Dance Center, NY
37.Stars Dance Studio, FL
38.Los Angeles Ballet Academy, CA
39.Fishback Studio of the Dance, NM
40.Anaheim Ballet Academy, CA
41.Mid-Atlantic Center for the Performing Arts, MD
42.MorningStar Dance Academy of Atlanta, GA
43.Americas Ballet School, FL
44.Colorado Ballet Academy, CO
45.Yuri Grigoriev School of Ballet, CA
46.Marina Almayeva School of Classical Ballet, TX
47.Steps On Broadway, NY
48.Festival Ballet Providence, RI
49.Summit Dance Shoppe, MN
50.Julianas Academy of Dance, MI
51.Coastal Dance Centre, SC
52.South Carolina Governors School for the Arts and Humanities, SC
53.Valentina Kozlova Dance Conservatory of New York, NY
54.Zamuel Ballet School, CO
55.Long Beach Ballet, CA
56.International Ballet Academy, NC
57.The Sarasota Cuban Ballet School Inc, FL
58.Academy of Russian Ballet, VA
59.Maryland Youth Ballet, MD
60.Peninsula School for Performing Arts, CA
61.Aika Ballet School, NY
62.Litchfield Dance Arts Academy, SC
63.Academy of Russian Classical Ballet, MI
64.All American Classical Ballet School, FL
65.First State Ballet Theatre Inc, DE
66.Westchester Dance Academy, NY
67.Olga Kresin Ballet School and Studio, PA
68.Elite Classical Coaching, TX
69.Joffrey Ballet Academy, NY
70.Russian Ballet Academy, AZ
71.Sarasota Ballet School, FL
72.Littleton Ballet Academy, CO
73.Joffrey Academy of Dance, IL
74.Columbia Conservatory of Dance, SC
75.Dance Industry Performing Arts Center, TX
76.Art Ballet Academy, TX
77.Florida School for Dance Education, FL
78.Pittsburgh Ballet House, PA
79.Cincinnati Ballet Otto M. Budig Academy, OH
80.Ballet Arte Academy of Classical Ballet, CA
81.Columbus Youth Ballet, OH
82.Ballet Tech Ohio, OH
83.The Ballet Clinic, AZ
84.Russian Ballet Academy of Indiana, IN
85.Dancing Arts Center, MA
86.Burbank Dance Academy, CA
87.Hudson Conservatory of Ballet, OH
88.Menlo Park Academy of Dance, CA
89.School of Classical Ballet & Dance, IA
90.Emerald Ballet Theatre, WA
91.Artistry in Motion Performing Arts Center, TX
92.North County Academy of Dance, CA
93.Ohio Conservatory of Ballet, OH

Figure S5: **Ranking of ballet schools.** The list contain the top 5% most prestigious U.S. ballet academies from the network-based ranking.

S3 YAGP Alumni

S3.1 Students' achievement

We quantify students' performance in the competition by means of the number of awards and the times they are listed as top students by the YAGP. We use these measures as a proxy of performance in ballet. We compute the following metrics:

i. *Student's total competitions, T_i*: the number of semi-final competitions as a top student. Multiple participation in the competition per year are possible, thus T_i denotes only competitions in the semi-finals.

ii. *Student's total number of awards, A_i*: the sum of awards a each student i obtains per competition j, considering the medals earned in each category (c = classical, m = contemporary), and the *Grand Prix* (g). Then, $A_i = A_{ic} + A_{im} + A_{ig}$, where for $A_{ic} = \sum_{j=1}^{T_i} a_{ij}$, $a_{ij} = 1$ when a dancer was awarded a medal in the classical category for the competitions j up to T_i for all semi-final competitions in C, and $a_{ij} = 0$ meaning that the student did not obtain a medal. The notation is the same for all possible awards in c, m, and g. One student cannot win more than one medal per category in each competition, then the possible number of awards in a given competition would be $A_{ij} = \{0, 1, 2, 3\}$. In a similar fashion, competition medals per student are counted by type (gold, silver, bronze).

iii. *Ratio of awards per student, R_i*: the ratio of obtained awards derived from the number of competitors ranked as top 12. The ratio of awards for students is given by $R_i = \frac{A_i}{T_i}$ and averages the awards obtained per student in each competition. Similarly to schools' ratio of awards, $R = 0$ indicates that no awards were obtained, $R = (0, 1)$ indicates less than one award per student, $R = 1$ gives an even relation between the number of awards and student (e.g. one student won one award every time he was listed as top 12), and $R > 1$ for those with more than one award per student in top 12. We label each group as 'no medals', 'under-achiever', 'break-even', and 'high-achiever', respectively for each ratio group mentioned above.

In Figure S6A, we observe the power-law distribution of students' number of awards, A_i. This indicates that only a few students win multiple awards, while the majority can achieve at least one medal. This distribution of awards is consistent with the one observed in the number of awards per school (Figure S2A). Most students (75%, 4824 students) have a success ratio lower than one, indicating that they win less than one award per competition. From this fraction, 3642 students (57% of all) obtained 'no medals', $R_i = 0$, even though they were listed in the top 12. Figure S6B shows that, from those who obtained at least one medal, a 18.5% (1182

students, in blue) are 'under-achievers', $R_i = (0, 1)$, meaning that they obtained less awards than the number of times they were listed in the top 12 ($A_i < T_i$). Conversely, a very small fraction of students (4.8%, 309 students, in red) are 'high-achievers', characterized by a high success ratio, $R_i > 1$, with more awards than the number of times they were listed in the top 12 ($A_i > T_i$). Only 19.7% of students (1260 students, in yellow) obtained a 'break-even' success ratio ($R_i = 1$). One would expect that the number of awards per student (A_i) strongly correlates with the total times in the top 12 (T_i), indicating that awards accumulate as students participate in the competition. Such relationship is moderate (Pearson's corr. coefficient=0.68), suggesting that being listed in the top 12 multiple times is not a strong but a mild predictor of the total number of awards. The majority of students (3511 students, 55%) have only one reported competition in the top 12 ($T_i = 1$), but we see in Figure S6D that all groups by ratio of awards have an exponential distribution respect to T_i. This indicates that there is a general pattern of variability in T_i that is independent of students' success ratio, S_i and also illustrates that the awards are actually scarce, even among top contenders.

To explore the distribution of awards, we compute the observed probability of obtaining an award, $P(\Lambda)$, where Λ represents each award by their implied rank. Such implied rank Λ is for semi-finals (SF): no medals, Bronze, Silver, Gold, Grand Prix; and for the finals (F): no medals, Bronze, Silver, Gold, Grand Prix (this rank does not assume the award value given by individuals). We divide the total number of awards per rank by the total number of top 12 students in the competition pool. Figure S6C shows that YAGP competitors have a 0.62 probability of being positioned in the top 12 without winning an award, while winning a competition medal in the semi-finals has about 0.1 of probability for gold, silver, and bronze. Interestingly, the probability of winning the *Grand Prix* in the semi-finals is rather low (0.03) but higher than being in the top 12 of the finals without obtaining an award (0.017 probability). Winning any competition medal in the YAGP finals has a very small probability ($Pr(\Lambda = \{\text{F: bronze, silver, gold}\}) < 0.005$), and the probability of winning the Grand Prix at this competition level is even smaller (0.002). Taking all together, our findings draw a picture of the level of competitiveness that YAGP competitors face and shows how repeated competitions do not imply a higher success ratio, even among top contenders, and raise the question about what factors different from persistence –and accumulated practice/experience– can determine dancers to be recognized by the jury in the YAGP competition venues.

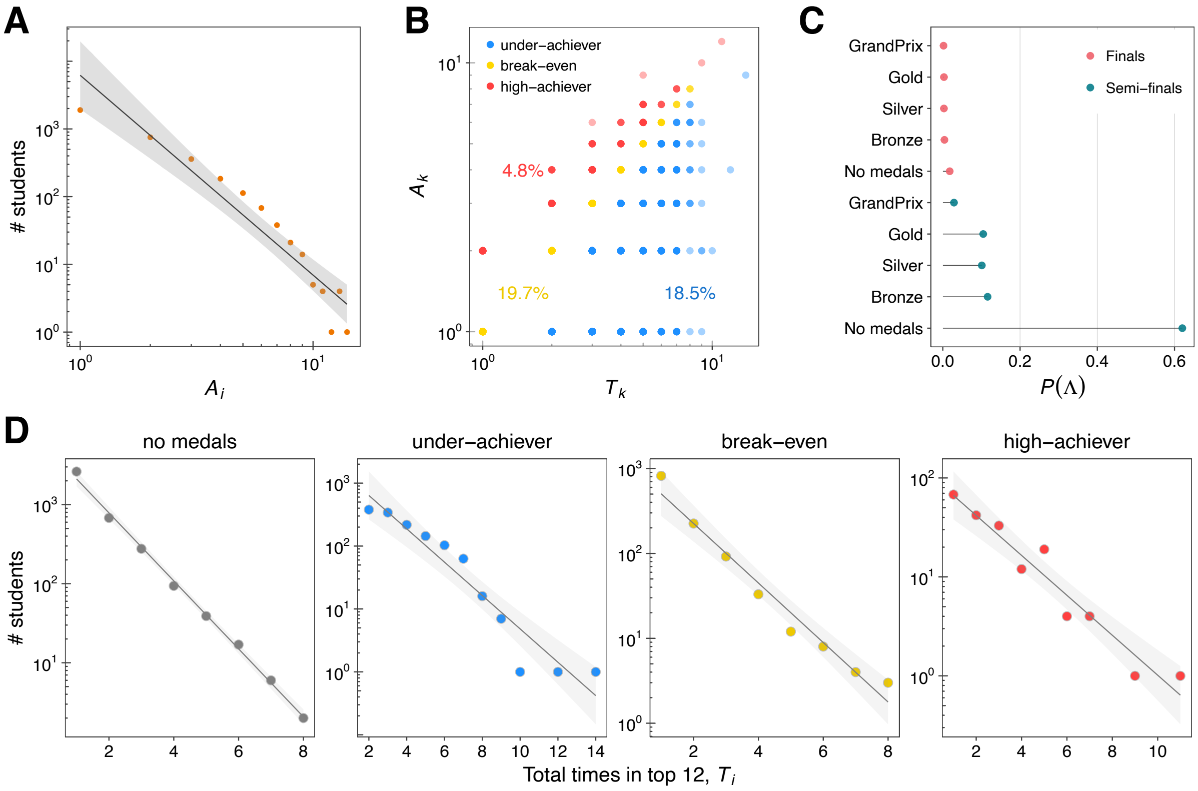

Figure S6: **Awards of ballet students. A** shows the count of the number number of awards per student A_i, with a power-law distribution. This indicates that most students obtained only one award, while only a few obtained more than 10 awards. **B** shows the percentage of students by group of success ratio, R_i, obtained from dividing the number of awards by the number years they positioned as top student. Taken together, 'no medals' ($R_i = 0$, in gray) and 'under-achievers' ($R_i = (0, 1)$, in cyan) represent the 75.3% of the total number of competitors (6393 students), meaning that most competitors win less than one award per top positioning. About 19.7% of students (1260 students) wins one award per top positioning ($R_i = 1$, in yellow), while only 4.8% of students (309 students) obtains more than one award per time they position as top student. **C** shows the probability of obtaining awards by rank for the finals/semi-finals in orange/blue. We see that a most top students do not obtain an award, and the probability of being awarded decreases as the rank increases, emphasizing scarcity in high rank awards (i.e. Finals). **D** shows the count of students and their number of competitions, T_i by their ratio of awards group, R_i, and demonstrates that all groups display an exponential distribution, suggesting that there is a general pattern of variability in the number of awards obtained per student disregarding of the number of semi-finals competitions.

S3.2 Students' success

Table S2 shows four variations of our base model. Model 1 corresponds to model described in Eq. 2. In model 2, we test for the potential effect of advancing to the competition finals by adding a dummy variable for being a finalist ($F_i = 1$) or not ($F_i = 0$). Models 1 and 2 are discussed in the results section.

In model 3, we examine the role of an affiliation for a job placement. Here, we replace the measure of school's prestige with a dummy variable for being affiliated ($D_i = 1$) or being an independent competitor ($D_i = 0$). This model shows no statistical effect for attending a school versus being an independent competitor, suggesting that there is no statistical difference of being affiliated or not regarding the chances of obtaining a job placement. Separately, in model 4 we explore the effect of total competition medals and observe that winning any medal has a significant but small positive effect on the chances of obtaining a job placement, increasing only by 49%.

In addition, we check for robustness of our treatment effect analysis on being affiliated to a top school, and observe that when we use the top 10% of schools (148 schools, 4217 controls, 3501 treated), the chances of job placement increase by 43% ($p = 0.0015$).

In sum, our analyses show that school prestige has a robust and significant positive effect on dancers' job placement, a comparable effect respect to being a finalist, but more importantly, that even when performance is similar, there are social factors such as prestige driving the selection of dancers towards successful company positions.

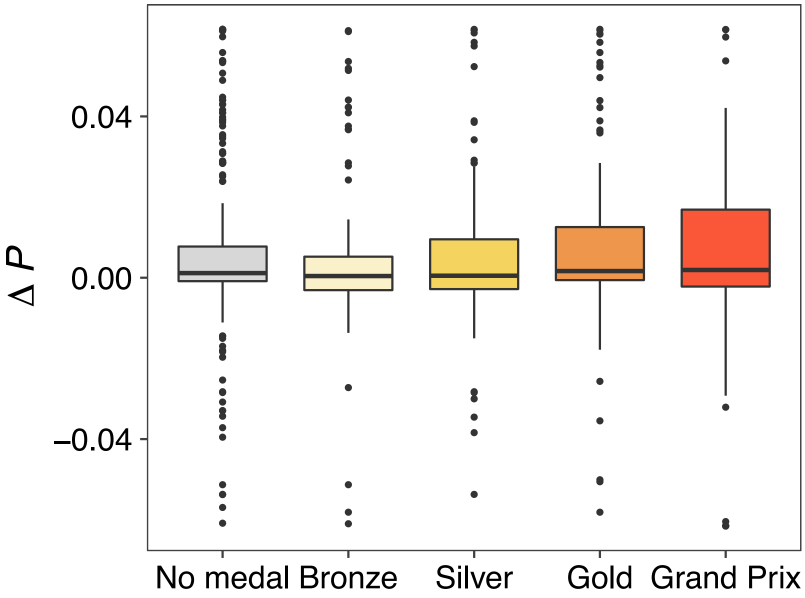

Figure S7: **Distribution of $\Delta Prestige$ from school change by award level.** $\Delta Prestige$ is calculated as $Prestige_{School_2} - Prestige_{School_1}$, hence a positive value represents moving to a more prestigious school. The observed median values of $\Delta Prestige$ across award levels obtained at School 1 show no statistical differences.

Table S2: **Model Coefficients for the Probability of Success.** Model coefficients labeled by p-value. Standard errors in parentheses.

	Dependent variable = Success S_i (job placement)			
	(1)	(2)	(3)	(4)
Affiliation D_i			0.319	
			(0.391)	
Prestige	1.201***	1.136***		1.203***
	(0.190)	(0.193)		(0.190)
Grand Prix	0.809***	0.515***	0.862***	0.831***
	(0.113)	(0.127)	(0.110)	(0.112)
Gold	0.45***	0.344***	0.448***	
	(0.091)	(0.093)	(0.088)	
Silver	0.45***	0.413**	0.472***	
	(0.091)	(0.091)	(0.089)	
Bronze	0.276**	0.258**	0.295**	
	(0.096)	(0.097)	(0.095)	
Total				0.403***
				(0.053)
Finalist F_i		1.0***		
		(0.184)		
Competitions	−0.238***	−0.208***	−0.234**	−0.244***
	(0.058)	(0.058)	(0.059)	(0.058)
Gender: male	0.754***	0.756***	0.915***	0.751***
	(0.121)	(0.121)	(0.116)	(0.121)
Constant	−3.135***	−3.193***	−3.608***	−3.138***
	(0.111)	(0.112)	(0.393)	(0.111)
Note:		*$p < 0.1$; **$p < 0.05$; ***$p < 0.01$		
Observations	6393	6393	6475	6393
McFadden pseudo R^2	0.085	0.095	0.075	0.08
AIC	2640.3	2615.3	2911.5	2638.9
AUC	0.7327	0.7374	0.7286	0.7312

References

[1] Vincent D Blondel et al. "Fast unfolding of communities in large networks". In: *Journal of statistical mechanics: theory and experiment* 2008.10 (2008), P10008. DOI: 10.1088/1742-5468/2008/10/P10008.

[2] Stanley Wasserman and Katherine Faust. *Social network analysis: Methods and applications*. Structural Analysis in the Social Sciences. Cambridge University Press, 1994. ISBN: 9780521387071.

[3] Don H Shamblin. "Prestige and the sociology establishment". In: *The American Sociologist* (1970), pp. 154–156. URL: https://www.jstor.org/stable/27701605.

[4] Bernd Wegener. "Concepts and measurement of prestige". In: *Annual review of sociology* (1992), pp. 253–280. DOI: 10.1146/annurev.so.18.080192.001345.

[5] Linton C Freeman. "A set of measures of centrality based on betweenness". In: *Sociometry* (1977), pp. 35–41. URL: https://www.jstor.org/stable/3033543.

[6] Gert Sabidussi. "The centrality index of a graph". In: *Psychometrika* 31.4 (Dec. 1, 1966), pp. 581–603. DOI: 10.1007/BF02289527.

[7] Linton C. Freeman. "Centrality in social networks conceptual clarification". In: *Social Networks* 1.3 (1978), pp. 215–239. ISSN: 0378-8733. DOI: 10.1016/0378-8733(78)90021-7.

[8] Phillip Bonacich. "Some unique properties of eigenvector centrality". In: *Social networks* 29.4 (2007), pp. 555–564. DOI: 10.1016/j.socnet.2007.04.002.

[9] *Dance Magazine: Pre-professional Program Guide 2022-23*. 2022. URL: https://www.dancemagazine.com/guide/dance-magazines-2022-23-pre-professional-program-guide/.

[10] David King. *A Ballet Education: The Blog*. URL: https://aballeteducation.com.

[11] Xavier Robin et al. "pROC: an open-source package for R and S+ to analyze and compare ROC curves". In: *BMC Bioinformatics* 12 (2011), p. 77. DOI: 10.1186/1471-2105-12-77.
